# Protective Effect of Quercetin on Sodium Iodate-Induced Retinal Apoptosis through the Reactive Oxygen Species-Mediated Mitochondrion-Dependent Pathway

**DOI:** 10.3390/ijms22084056

**Published:** 2021-04-14

**Authors:** Yuan-Yen Chang, Yi-Ju Lee, Min-Yen Hsu, Meilin Wang, Shang-Chun Tsou, Ching-Chung Chen, Jer-An Lin, Yai-Ping Hsiao, Hui-Wen Lin

**Affiliations:** 1 Department of Microbiology and Immunology, School of Medicine, Chung Shan Medical University, Taichung 40201, Taiwan; cyy0709@csmu.edu.tw (Y.-Y.C.); wml@csmu.edu.tw (M.W.); 2 Department of Medical Education, Chung Shan Medical University Hospital, Taichung 40201, Taiwan; 3 Department of Pathology, Chung Shan Medical University Hospital, Chung Shan Medical University, Taichung 40201, Taiwan; jasmine.lyl@gmail.com; 4 Department of Ophthalmology, Chung Shan Medical University Hospital, Taichung 40201, Taiwan; my.scott.hsu@gmail.com (M.-Y.H.); amy1234575@gmail.com (Y.-P.H.); 5 School of Medicine, Chung Shan Medical University, Taichung 40201, Taiwan; 6 Biotechnology Center, National Chung Hsing University, Taichung 40201, Taiwan; 7 Department of Biomedical Sciences, Chung Shan Medical University, Taichung 40201, Taiwan; eq7bie5d@gmail.com; 8 Department of Optometry, Asia University, Taichung 41354, Taiwan; art@asia.edu.tw; 9 Graduate Institute of Food Safety, National Chung Hsing University, Taichung 40201, Taiwan; lja@nchu.edu.tw; 10 Department of Medical Research, China Medical University Hospital, China Medical University, Taichung 40402, Taiwan

**Keywords:** age-related macular degeneration, sodium iodate, human retinal pigment epithelium, quercetin, apoptosis, mitochondrial membrane potential

## Abstract

Age-related macular degeneration (AMD) leads to gradual central vision loss and is the third leading cause of irreversible blindness worldwide. The underlying mechanisms for this progressive neurodegenerative disease remain unclear and there is currently no preventive treatment for dry AMD. Sodium iodate (NaIO_3_) has been reported to induce AMD-like retinal pathology in mice. We established a mouse model for AMD to evaluate the effects of quercetin on NaIO_3_-induced retinal apoptosis, and to investigate the pertinent underlying mechanisms. Our in vitro results indicated that quercetin protected human retinal pigment epithelium (ARPE-19) cells from NaIO_3_-induced apoptosis by inhibiting reactive oxygen species production and loss of mitochondrial membrane potential as detected by Annexin V-FITC/PI flow cytometry. We also evaluated the relative expression of proteins in the apoptosis pathway. Quercetin downregulated the protein expressions of Bax, cleaved caspase-3, and cleaved PARP and upregulated the expression of Bcl-2 through reduced PI3K and pAKT expressions. Furthermore, our in vivo results indicated that quercetin improved retinal deformation and increased the thickness of both the outer nuclear layer and inner nuclear layer, whereas the expression of caspase-3 was inhibited. Taken together, these results demonstrate that quercetin could protect retinal pigment epithelium and the retina from NaIO_3_-induced cell apoptosis via reactive oxygen species-mediated mitochondrial dysfunction, involving the PI3K/AKT signaling pathway. This suggests that quercetin has the potential to prevent and delay AMD and other retinal diseases involving NaIO_3_-mediated apoptosis.

## 1. Introduction

Age-related macular degeneration (AMD) is a progressive neurodegenerative condition and the leading cause of blurry vision and blindness in the elderly population of industrialized nations. AMD can be divided into early, intermediate and late AMD. Late AMD is further classified into dry (atrophic) and wet (neovascular) subtypes. Dry AMD is more common, accounting for approximately 90% of all cases, and 10% of dry AMD cases progress to wet AMD [1]. The primary treatment for wet AMD is the administration of antivascular endothelial growth factor, but at present, no effective treatment strategies exist for dry AMD. This is because the pathogenesis of dry AMD is complicated and involves various cellular pathways [2]. Dry AMD is characterized by drusen and widespread retinal pigment epithelium (RPE) degeneration.

Risk factors associated with AMD include age, oxidative stress, genetic factors, chronic inflammation, and race [3,4,5,6,7,8]. The development of AMD can be primarily attributed to exposure to excessive reactive oxygen species (ROS) [9,10,11,12]. ROS overproduction destroys the activity of RPE cells, including antioxidant reactions, metabolic processes, and lysosomal degradative functions, eventually leading to apoptosis [13].

Chew et al. reported that antioxidant vitamins and zinc supplements can decelerate AMD development [14]. The pathological mechanism underlying AMD has not yet been completely elucidated, and the condition remains untreatable at present. Sodium iodate (NaIO_3_), an oxidative toxic agent, causes selective RPE cell damage and can reportedly serve as a reproducible in vitro and in vivo model of AMD [15,16,17,18]. NaIO_3_ at a concentration of ≥10 mM activates caspase 3/7/8-dependent apoptosis and caspase-independent cell necroptosis, leading to the apoptosis of RPE cells [19,20,21,22].

Hwang et al. (2019) reported that NaIO_3_ induced cytosolic ROS production but not mitochondrial ROS production; furthermore, it activated ERK, p38, JNK, and protein kinase B (AKT) signaling pathways [23]. They also found that cytosolic ROS-dependent p38 and JNK activation led to the death of NaIO_3_-treated ARPE-19 cells (human retinal pigment epithelial cells), whereas cytosolic ROS-mediated autophagy and mitochondrial dynamic balance contributed to cell survival. Moreover, their data suggested that ROS, AKT, and ERK signaling pathways played a role in pentraxin 3 (also known as the tumor necrosis factor-α-stimulated gene) production in response to NaIO_3_ in ARPE-19 cells.

Quercetin is a type of flavonoid found in plant products. It has numerous pharmacological applications and possesses antioxidant, neuroprotective, anti-inflammatory, antiangiogenic, and antiapoptotic properties [24,25]. Many studies have described the antioxidant activities of quercetin, including its ability to reduce and inhibit the damage caused by oxidative stress, both in vitro and in vivo [26,27]. Sharmila et al. (2014) confirmed that quercetin inhibits insulin-like growth factor receptor-1, AKT, androgen receptor (AR), cell proliferation, and antiapoptotic proteins in an animal model of prostate cancer [28]. Interestingly, quercetin has been reported to regulate antioxidant levels, thus protecting nerves, the brain, and some human cells from oxidation-induced damage. Furthermore, Ossola et al. (2009) found that the quercetin glycoside can cross the blood–brain barrier and has more effective protective activity compared with rutin and isoquercetin glycosides [29]. Weng et al. (2017) demonstrated that quercetin protected ARPE-19 cells from H_2_O_2_-induced cell damage by activating the Nrf2 signaling pathway, inhibiting endoplasmic reticulum stress, and targeting antiapoptotic proteins [30]. However, to the best of our knowledge, the protective effects of quercetin via stimulation of phosphatidylinositol 3-kinase (PI3K) expression and downstream signaling molecules and the NaIO_3_-induced regulation of ROS and apoptotic proteins in ARPE-19 cells and AMD animal models have not yet been determined.

## 2. Materials and Methods

### 2.1. Cell Culture

The human retinal epithelial cell line ARPE-19 (at passage 27, product CRL-2302, American Type Culture Collection, ATCC, Manassas, VA, USA) was maintained in Dulbecco’s modified Eagle’s medium/F12 Ham nutrient mixture (HyClone, Logan, UT, USA) containing 10% fetal bovine serum (Gibco) at 37 °C and 5% CO_2_.

### 2.2. Cell Viability Assay

ARPE-19 cells (1.5 × 10^5^ cells/well) were seeded into 24-well plates at 1 mL volume and incubated at 37˚C for 24 h. The culture medium was subsequently replaced by a medium containing various doses of quercetin (0, 1.25, 2.5, 5, 10, and 20 µM) alone or with a co-treatment of NaIO_3_. After 24 h, 0.5 mL culture medium containing 10 µL Cell Counting Kit-8 reagent (Dojindo Molecular Technologies, Kumamoto, Japan) was added to the cells in each well and they were incubated at 37 °C for 1–4 h. The absorbance of each well was then measured at 450 nm using a ELISA reader (Multiskan Spectrum, Thermo Co., Vantaa, Finland).

### 2.3. Measurement of Intracellular ROS and H_2_O_2_ Production

Intracellular ROS production was determined by measuring the oxidation of 2′,7′ dichlorofluorescein diacetate (DCFH-DA) to the highly fluorescent compound 2′,7′-dichlorofluorescein (DCF). ARPE-19 cells were cultured in 12-well plates and pretreated with different concentrations (1.25, 2.5, and 5 μM) of quercetin for 1.5 h, followed by incubation with 6 mM NaIO_3_ at 37 °C for 15 h. Subsequently, 10 µM DCFH-DA was added to the culture medium, and the reaction was allowed to proceed at 37 °C for 30 min. The cells were then washed with PBS and collected for flow cytometry (BD Biosciences, San Jose, CA, USA). Data analysis was performed using CellQuest.

### 2.4. Measurement of Mitochondrial Damage

For the analysis of mitochondrial status, the cells were incubated with JC-1 dye (2 µg/mL; Cayman Chemical, Ann Arbor, MN, USA) for 50 min, then 2 μL Hoechst33342, a DNA-specific fluorescent dye, was added and the cells were incubated in darkness at 37 °C for 10 min. The stained cells were then observed under a fluorescence microscope. JC-1 accumulates in mitochondria and appears as a red/orange fluorescence (590 nm) in healthy organelles, however, when depolarized it appears as a green fluorescence (530 nm). Fluorescence images of the cells were recorded and the relative intensities of green/red JC-1 fluorescence were quantified using Image J Software (U.S. National Institutes of Health, Bethesda, MD, USA).

### 2.5. Measurements of Antioxidative Capacities and H_2_O_2_ Production

The activities of superoxide dismutase (SOD), catalase (CAT), and reduced glutathione (GSH) were analyzed using assay kits from Cayman according to the manufacturer’s instructions (Cat. 706002, 707002 and 703002, Cayman, Ann Arbor, MI, USA).

The H_2_O_2_ production in the culture medium was determined using a Biovision assay kit (Biovision Research Products, Milpitas, CA, USA) following the manufacturer’s instructions. The absorbance was measured at 570 nm with an ELISA reader.

### 2.6. Western Blot Analysis

ARPE-19 cells from different experimental conditions were lysed in a lysis buffer [10 mM Tris, pH 7.5 (Sigma, St. Louis, MO, USA), 1 mM EDTA, and 0.1% Triton X-100 (Sigma, St. Louis, MO, USA). All samples were then electrophoresed on 10% SDS-PAGE, and the separated proteins were transferred onto a PVDF membrane. The membrane was incubated overnight with Bcl, Bax, caspase-3, cleaved caspase-3, cleaved PARP, PI3K p100, p-AKT, and GAPDH or β-tubulin primary antibodies. Band intensities were measured using AlphaImager 2200 software (Alpha Innotech Co., San Leandro, CA, USA).

### 2.7. Animal Model

Forty-two-week-old BABL/c mice were purchased from Asia University Animal Care and Use Committee (IACVC No. 107-a51a-20, 01 August 2019) and housed in standard cages with a 12:12-h light–dark cycle. The mice were randomly divided into three groups, with each group containing ten mice:

1. Mock group: Animals pretreated with an intraperitoneal (IP) injection of PBS and then a single intravenous (IV) injection of PBS.
2. Vehicle-treated group: Animals pretreated with an IP injection of PBS and then a single IV injection of 40 mg/kg NaIO_3_ [31].
3. Experimental group: Animals pretreated with an IP injection of 100 mg/kg quercetin and then a single IV injection of 40 mg/kg NaIO_3_.

### 2.8. Histology and Immunohistochemistry

The eyeballs were fixed in Davidson’s solution (containing 10% formalin, 10% glacial acetic acid, and 4% formaldehyde) for 3 days [32]. Paraffin-embedded sections (5 μm) were obtained, stained with hematoxylin and eosin (H&E), and photographed using an optical microscope (Olympus Optical, Tokyo, Japan). The thicknesses of the whole retina, outer nuclear layer (ONL), and inner nuclear layer (INL) were measured at a distance of between 600 μm and 900 μm from the optic nerve along the superior and inferior hemiretina. Data from six random sites were averaged for each eye.

Immunohistochemical staining was performed using a BondMax automated slide staining system (Vision BioSystems Ltd., Newcastle Upon Tyne, UK). The sections were subjected to antibody cleaved caspase-3 (1:100, Cell Signaling Technology, Danvers, MA, USA) and then photographed using an optical microscope (Olympus Optical). Caspase-3 staining was quantified using the Image J Immunohistochemistry Toolbox (National Institute of Health, Starkville, MD, USA).

### 2.9. Retinal Imaging

Optical coherence tomography (OCT) was performed using RTVue XR Avanti with AngioVue (Optovue Inc, Fremont, CA, USA). Briefly, OCT of a certain region of the retina was performed repeatedly, and the resultant scans were examined for changes. Additional information pertaining to OCT can be found in two previous studies [33,34].

### 2.10. Statistical Analysis

The current study was conducted using a completely randomized design. When a significant difference (*p* < 0.05) was detected among groups by one-way analysis of variance (ANOVA), differences among the treatments were further tested using the least significant difference (LSD) test. All statistical analyses were performed using Statistical Analysis Software (SAS Institute Inc., Cary, NC, USA, 2002).

## 3. Results

### 3.1. Effect of Quercetin Pretreatment on the Viability and NaIO_3-_Induced Apoptosis of ARPE-19 Cells

**Figure 1:**
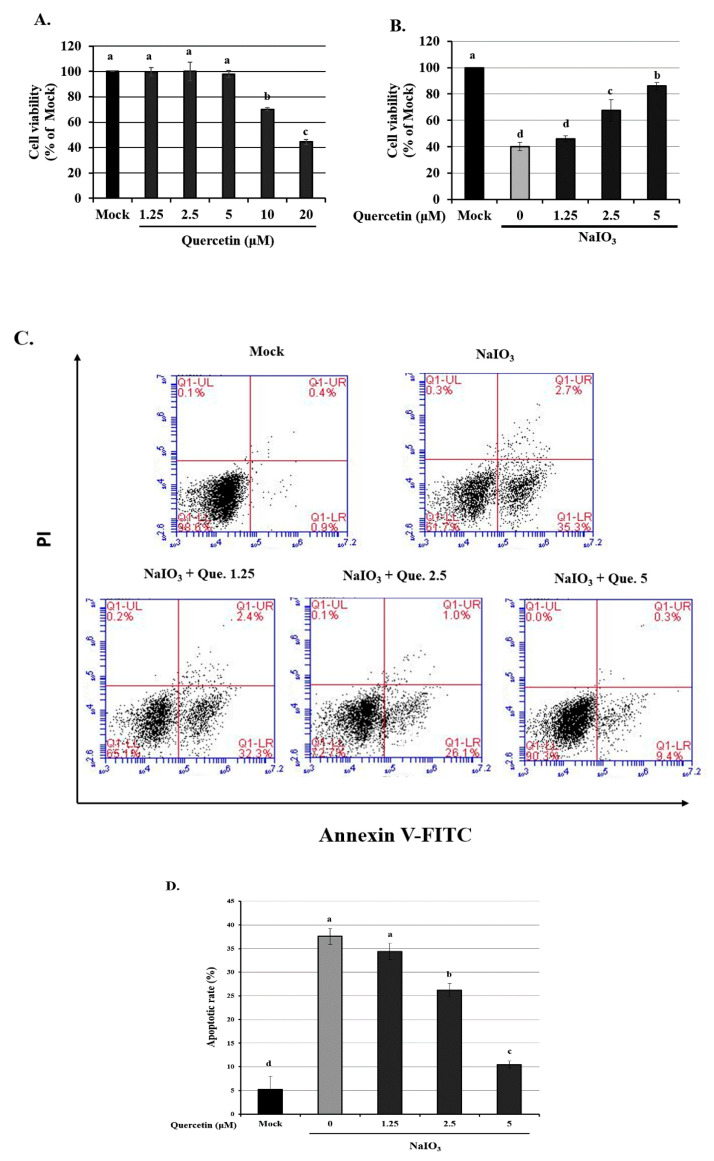
Cell viability of ARPE-19 cells treated with quercetin only or quercetin plus NaIO_3_. (**A**) ARPE-19 cells were treated with various concentrations of quercetin (1.25, 2.5 and 5 μM) for 24 h, and the cell viability was measured using a CCK-8 assay. (**B**) ARPE-19 cells were pretreated with various concentrations of quercetin (1.25, 2.5 and 5 μM) for 1.5 h and then with NaIO_3_ (6 mM) for 24 h, before cell viability was measured using the CCK-8 assay. (**C**) ARPE-19 cells were pretreated with various concentrations (1.25, 2.5 and 5 μM) of quercetin for 1.5 h before treatment with NaIO_3_ (6 mM) for 15 h. ARPE-19 cells were then detected by flow cytometry after staining with both Annexin V-FITC and PI for 30 min. (**D**) The percentage of apoptotic cells in each treatment group was quantified. Values represent the mean ± SD (*n* = 3). Data bars without letters in common (a–d) indicate a significant difference (*p* < 0.05).

To determine whether quercetin is toxic to ARPE-19 cells, we exposed them to various concentrations of quercetin and then evaluated the cell viability. The cell survival rate was unaffected following treatment of cells with <5 µM quercetin (Figure 1A). However, treatment with 10–20 µM quercetin significantly reduced the cell viability, with effects being significantly different from the untreated controls (0 µM). For this reason, 1.25, 2.5, and 5 μM quercetin were used for all subsequent experiments.

We also determined the viability of ARPE-19 cells, which were treated with various concentrations of quercetin (1.25, 2.5, and 5 µM) and 6 mM NaIO_3_ (Figure 1B). Cell viability significantly decreased from 100% in the mock group to 70% in the vehicle-treated group. All tested concentrations (1.25, 2.5, and 5 µM) of quercetin significantly increased the cell viability when ARPE-19 cells were treated with 6 mM NaIO_3_.

Apoptosis was detected in NaIO_3_-treated ARPE-19 cells using an Annexin V-FITC/PI apoptosis detection kit and flow cytometry. The rates of apoptosis following pretreatment with 1.25, 2.5 and 5 µM quercetin for 1.5 h before treatment with 6 mM NaIO_3_, were 34.6 ± 2.1, 26.9 ± 3.4 and 11.4 ± 2.9%, respectively, and were significantly decreased compared with the NaIO_3_ only treated group (38.1 ± 1.5%; *p* < 0.05) (Figure 1C,D). These results indicated that quercetin decreased NaIO_3_-induced cell death.

### 3.2. Quercetin Reduced the Expression of Intracellular ROS and H_2_O_2_ Production

**Figure 2:**
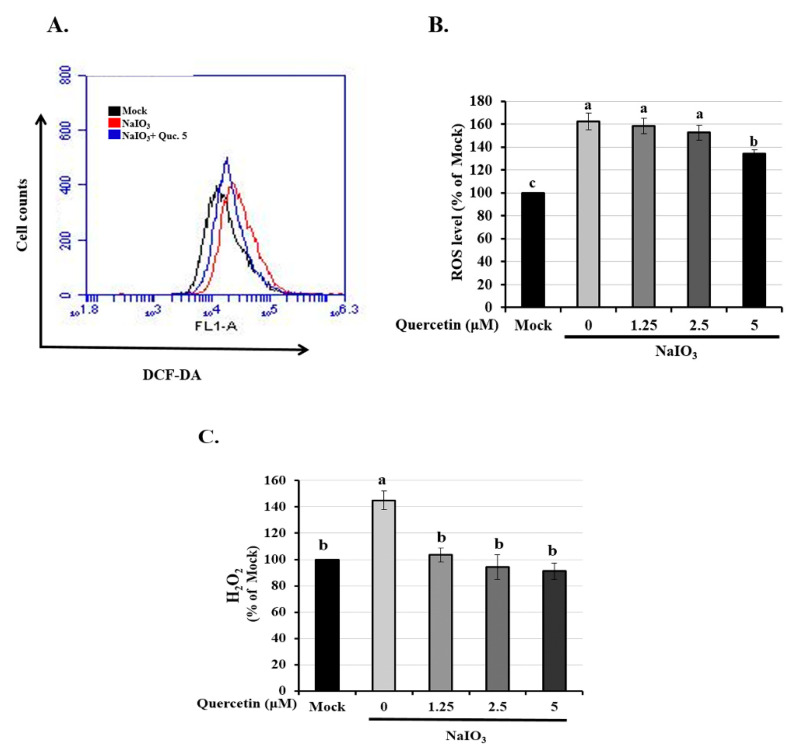
Effect of quercetin on NaIO_3_-mediated ROS generation in ARPE-19 cells. ARPE-19 cells were pretreated with different concentrations of quercetin (1.25, 2.5, and 5 μM) for 1.5 h and then treated with NaIO_3_ (6 mM) for 15 h. (**A**,**B**) Cells were labeled with the fluorescent probe, 2′,7′ dichlorodihydrofluorescein diacetate, the intracellular ROS levels were quantitatively analyzed and the mean fluorescence intensity was calculated using flow cytometry. (**C**) ARPE-19 cells were pretreated with different concentrations of quercetin (1.25, 2.5, and 5 μM) for 1.5 h and then treated with NaIO3 (6 mM) for 24 h. The amount of H_2_O_2_ were measured by commercial assay kits. Values represent the mean ± SD (*n* = 3). Data bars without letters in common (a–c) indicate a significant difference (*p* < 0.05).

ROS is closely associated with oxidative stress, which can lead to cell apoptosis [34]. Oxidative stress induced by NaIO_3_ has been reported to cause apoptosis and autophagy [35]. Therefore, we assessed the effects of intracellular and mitochondrial ROS on NaIO_3-_induced ARPE-19 cell death. After treatment with NaIO_3_ for 15 h, the cells were labeled with DCFH-DA (an intracellular ROS probe) and analyzed through flow cytometry. As illustrated in Figure 2A,B, the mean ROS-associated fluorescence intensity in the NaIO_3_-treated group was significantly higher than in the mock group. Compared with the NaIO_3_-treated group, pretreatment with quercetin attenuated the NaIO_3_-induced intracellular ROS levels in the ARPE-19 cells (Figure 2B). We then measured the level of hydrogen peroxide (H_2_O_2_) again using a commercial kit, and the results again showed that the increased expression of H_2_O_2_ in the NaIO_3_-induced group was significantly eliminated after treatment with quercetin (1.25–5 μM) (Figure 2C).

### 3.3. Quercetin Inhibited the Reduction of Mitochondrial Membrane Potential (∆Ψm) via Modulated the Activity of Anti-Oxidants

**Figure 3:**
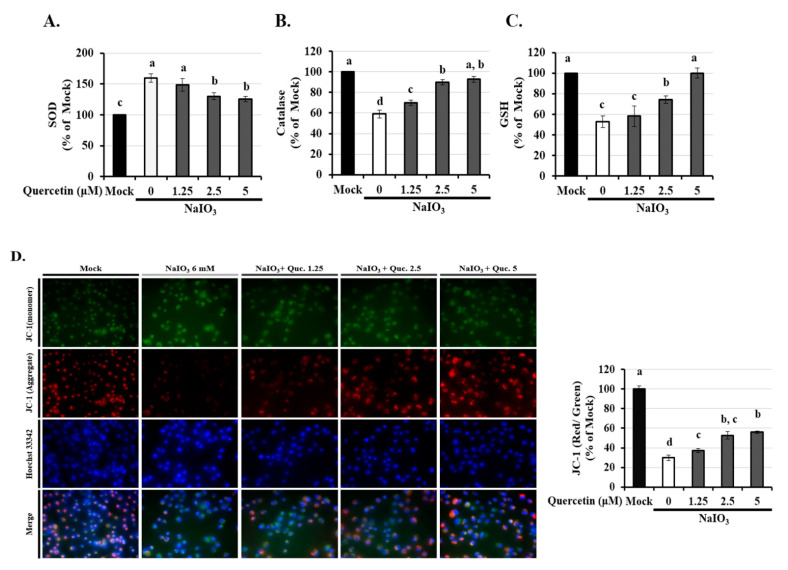
Effects of quercetin on antioxidant activity and mitochondrial dysfunction in ARPE-19 cells. ARPE-19 cells were pretreated with different concentrations of quercetin (1.25, 2.5, and 5 μM) for 1.5 h and then treated with NaIO3 (6 mM) for 15 h. The levels of (**A**) superoxide (SOD), (**B**) catalase, and (**C**) glutathione (GSH) were quantitatively analyzed, and the mean fluorescence intensity was calculated using flow cytometry. (**D**) Mitochondrial membrane potential was measured using the fluorescent probe JC-1. Loss of mitochondrial membrane depolarization (ΔΨm) was demonstrated by the change in JC-1 fluorescence from red (JC-1 aggregates) to green (JC-1 monomers). The fluorescent intensity ratio of JC-1 aggregates to monomers in treated cells. Values represent the mean ± SD (*n* = 3). Data bars without letters in common (a–d) indicate a significant difference (*p* < 0.05).

It is well known that the activity of glutathione (GSH), superoxide (SOD), and catalase protects cells against ROS-induced oxidative damage [13]. As shown in Figure 3A–C, the expressions of anti-oxidants except SOD were dramatically decreased in the NaIO_3_-induced group. Quercetin (2.5 and 5 µM) treatment significantly reversed the reduced levels of catalase and GSH and the increased level of SOD, indicating that quercetin could modulate the activity of anti-oxidants. Since mitochondrial membrane integrity is sensitive to cellular ROS, we assessed disruption of mitochondrial membrane potential in ARPE-19 cells after quercetin pre-treatment. ARPE-19 cells exposed to NaIO_3_ for 15 h had a decreased ΔΨm, suggesting mitochondrial disruption, as indicated by a decrease in the red/green fluorescence intensity ratio (Figure 3D). However, treatment with 2.5 and 5 µM quercetin significantly increased the ΔΨm. These results indicated that quercetin could protect cells from NaIO_3_-mediated ROS injury by maintaining ROS levels and the ΔΨm.

### 3.4. Effect of Quercetin on the Endogenous NaIO_3_-Induced Apoptotic Pathway

**Figure 4:**
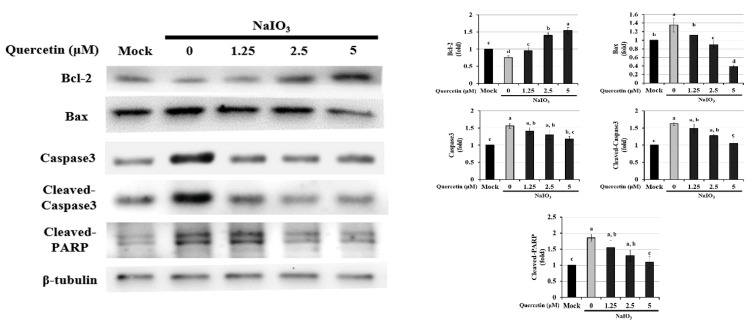
Western blotting was performed to assess the effects of quercetin on the expression of Bcl-2, Bax, caspase-3, cleaved caspase-3, and cleaved PARP in NaIO_3_-treated ARPE-19 cells. ARPE-19 cells were pretreated with different concentrations of quercetin (1.25, 2.5, and 5 μM) for 1.5 h and then treated with NaIO_3_ (6 mM) for 24 h. Data were normalized to β-tubulin. Values represent the mean ± SD (*n* = 3). Data bars without letters in common (a–d) indicate a significant difference (*p* < 0.05).

Activation of caspase-3 and a decrease in Bcl-2 are typical markers of apoptosis; Bax is also an integral part of this apoptotic pathway [36,37,38]. We found that NaIO_3_ induced apoptosis. To investigate how quercetin protects ARPE-19 cells from NaIO_3_-induced apoptosis, we used Western blotting to evaluate the effect of quercetin on the expression of apoptosis-related proteins (Bcl-2, Bax, caspase-3, cleaved caspase-3, and cleaved PARP) (Figure 4). Compared with the mock group, exposure to 6 mM NaIO_3_ led to a lower level of Bcl-2 and higher levels of Bax, caspase-3, cleaved caspase-3, and cleaved PARP. However, treatment with 5 µM quercetin significantly reduced the expression levels of Bax, cleaved caspase-3, and cleaved PARP, compared with the NaIO_3_-treated group. In addition, quercetin increased the protein expression of Bcl-2 compared with the mock group. These results indicated that quercetin inhibited cell apoptosis, downregulated the protein expressions of Bax, cleaved caspase-3, and cleaved PARP, and upregulated the protein expression of Bcl-2 in NaIO_3_-treated ARPE-19 cells.

### 3.5. Quercetin Suppressed Apoptosis via the PI3K/AKT Signaling Pathway

The PI3K signaling pathway promotes cell survival and reportedly participates in apoptosis in the central nervous system. AKT, a serine/threonine protein kinase that is also known as protein kinase B, is the primary downstream effector of the PI3K signaling pathway [39]. In a recent study, Chan et al. (2019) reported that NaIO_3_ induced cytosolic ROS production by activating ERK, p38, JNK, and the AKT signaling pathway [36]. To determine whether the antiapoptotic effects of quercetin on NaIO_3_-treated ARPE-19 cells were mediated through the PI3K/AKT signaling pathway, we performed Western blotting to measure AKT phosphorylation.

**Figure 5:**
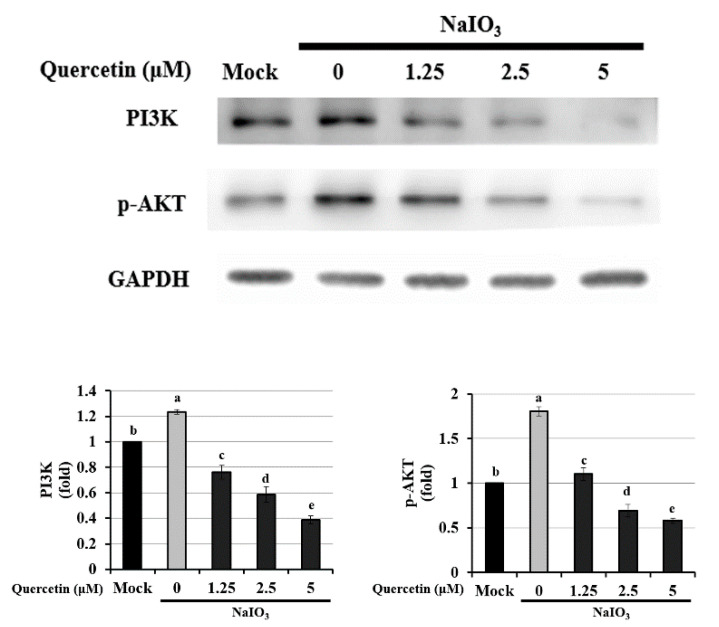
Western blotting to determine the expression of PI3K and p-AKT in NaIO_3_-treated ARPE-19 cells. ARPE-19 cells were pretreated with different concentrations of quercetin (1.25, 2.5, and 5 μM) for 1.5 h and then treated with NaIO_3_ (6 mM) for 24 h. Data were normalized to GAPDH. Values represent mean ± SD (*n* = 3). Data bars without letters in common (a–e) indicate a significant difference (*p* < 0.05).

As shown in Figure 5, the levels of PI3K and phosphorylated-AKT (p-AKT) were significantly higher in the NaIO_3_-treated group compared with the mock and experimental groups. This indicates that quercetin inhibited the PI3K/AKT signaling pathway and suggests that this pathway is involved in the underlying mechanism of apoptosis in NaIO_3_-treated ARPE-19 cells.

### 3.6. Protective Effects of Quercetin on Retinal Degeneration

To confirm the association between in vitro and in vivo experiments on retinal damage caused by NaIO_3_, we established a retinal degeneration mouse model. After 7 days of NaIO_3_ treatment, OCT was used to observe the thickness of the whole retina, INL and ONL, and the results were validated by histological analyses (H&E staining).

**Figure 6:**
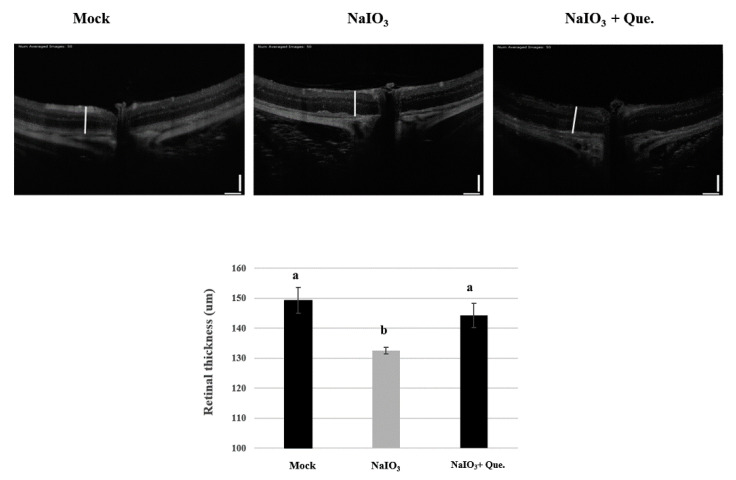
Preventive effects of quercetin on the retinal thickness of NaIO_3_-treated mice. Optical coherence tomography (OCT) was performed 7 days after NaIO_3_ treatment for all three study groups. The retinal degenerative changes are shown. Vertical bar = 100 μm and horizontal bar = 120 μm; white line represents retinal thickness. Values represent mean ± SD (*n* = 6). Data bars without letters in common (a,b) indicate a significant difference (*p* < 0.05).

OCT is a noninvasive technique based on optical reflectivity and it is able to analyze retinal thickness and structure to produce three-dimensional, cross-sectional, retinal images [34]. It has been clinically adopted as the standard method to observe structural changes associated with retinopathy. In the current study, the OCT images of day 7 NaIO_3_-treated mice showed that their total retinal thickness was significantly lower than the mice in the mock group (Figure 6). However, quercetin treatment was shown to effectively restore the retinal thickness.

**Figure 7:**
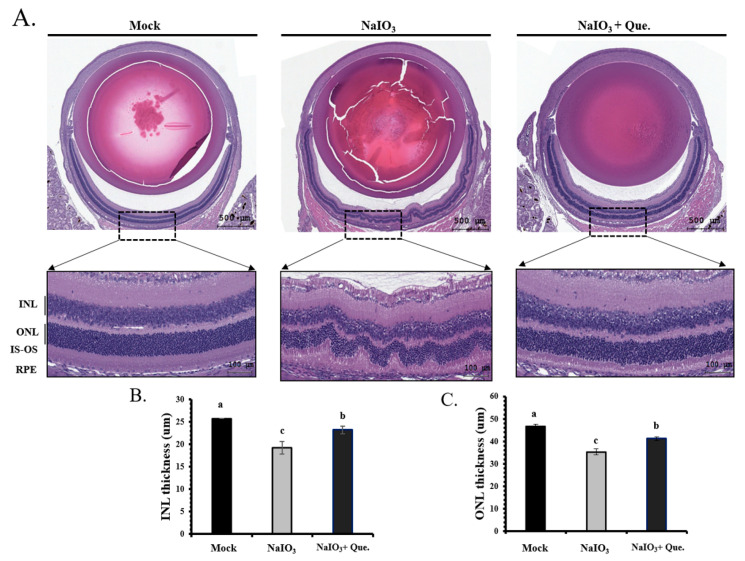
Protective effects of quercetin on retinal degeneration in NaIO_3_-treated mice. (**A**) Representative retinal sections (H&E staining) for the three groups from between 600 μm and 900 μm from the optic nerve along the superior and inferior hemiretina. ONL, outer nuclear layer; INL, inner nuclear layer; IS-OS, inner and outer segment of photoreceptor; RPE, retinal pigment epithelium. Scale bar = 50 μm. (**B**) INL and (**C**) ONL thickness were measured at six locations and the values were then averaged. Values represent the mean ± SD (*n* = 6). Data bars without letters in common (a–c) indicate a significant difference (*p* < 0.05).

Representative images are shown for the retinal sections from the three study groups (Figure 7A). The thicknesses of the total retina, INL and ONL were measured. In the NaIO_3_-treated mice, the thicknesses of the total retina, INL and ONL were significantly lower than in the mice from the mock group (*p* < 0.05). In addition, disruption between the inner segment and the outer segment of the photoreceptor (IS/OS) was found in NaIO_3_-treated mice (Figure 7A).

Quercetin treatment protected the retinal layers against loss and caused partial recovery of the IS/OS disruption (Figure 7A). INL and ONL thicknesses were also lower in the NaIO_3-_treated mice; however, the degeneration was almost alleviated via quercetin treatment (Figure 7B,C). The degree of retinal degeneration was quantified by measuring the ONL thickness. The number of ONL nuclei was counted to demonstrate the degeneration of the retinal layers. Treatment of NaIO_3_-treated mice with quercetin prevented the decrease in ONL thickness and nuclei counts. Overall, quercetin administration effectively prevented retinal degeneration caused by NaIO_3_.

### 3.7. Quercetin Reduced NaIO_3_-Induced Retinal Apoptosis in Mice

**Figure 8:**
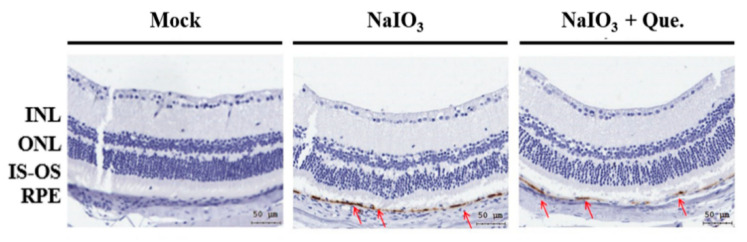
Effect of quercetin on NaIO_3_-induced retinal apoptosis in mice. After 7 days of NaIO_3_ treatment, immunohistochemical staining was performed for cleaved caspase-3 in all three study groups. The red arrows indicate cleaved caspase-3 in the retinal pigment epithelial (RPE) layers. The vehicle-treated NaIO_3_ showed a significantly higher expression of cleaved caspase-3, while the mock and experimental groups showed significantly lower expression of cleaved caspase-3. Cleaved caspase-3 is shown in brown, and the red arrows are within the position of the RPE layer.

We further explored the protective effects of quercetin on NaIO_3_-induced retinal cell injury. After 7 days of NaIO_3_ treatment, the mock, vehicle-treated, and experimental groups were subjected to immunohistochemical staining for cleaved caspase-3. Compared with the other two groups, the expression of cleaved caspase-3 was significantly higher in the vehicle-treated group (Figure 8), indicating that quercetin significantly reduced the production of NaIO_3_-induced cleaved caspase-3.

## 4. Discussion

AMD is the primary cause of blindness in the elderly population of developed countries. Millions of patients with AMD experience gradual vision loss year by year and no preventive treatment yet exists for decreasing dry AMD, which can progress to wet AMD [40,41,42]. Therefore, the identification of potential anti-AMD agents is extremely important. AMD is associated with several risk factors, and many of them are highly linked to increased ROS. The current literature also indicates that excessive ROS triggers AMD development, leading to apoptosis of RPE cells [13]. Previous in vivo and in vitro studies have reported that inhibitors of caspases and necroptotic signaling pathways inhibit NaIO_3_-induced RPE and photoreceptor cell death [23,35,43,44]. Chan et al. reported that NaIO_3_-induced AKT activation was partially dependent on ROS production, eventually leading to cell death [36]. In addition, blue light can induce apoptosis by increasing the cleaved form of caspase-3 and the Bax/Bcl-2 ratio in RPE cells [45,46]. PARP-1 plays a crucial role in DNA repair, replication, and cell death. PARP-1 cleavage is observed during apoptosis, which is induced by caspase-3 activation [36].

In order to investigate the effects of quercetin on the retina, an NaIO_3_-induced experimental model of AMD was established in vitro and in vivo. In this study, we found that NaIO_3_-induced ROS production caused apoptosis by upregulating the expressions of Bax, cleaved caspase-3, and cleaved PARP, and downregulating Bcl-2 expression through the PI3K/AKT signaling pathway in ARPE-19 cells (Figure 4 and Figure 5). In addition, we found that quercetin inhibited NaIO_3_-induced cell apoptosis by mediating PI3K/AKT inactivation, thereby downregulating the expressions of Bax, cleaved caspase-3, and cleaved PARP and upregulating Bcl-2 expression (Figure 4). Consistent with these findings, we found that quercetin attenuated NaIO_3_-induced increases in cleaved caspase-3 levels. Taken together, our findings suggest that the antiapoptotic effects of quercetin are mediated by the inhibition of apoptotic effectors. The results of this study showed that quercetin significantly protected ARPE-19 cells against NaIO_3_-induced toxicity, high ROS levels and apoptosis in vitro.

NaIO_3_-induced retinochoroidal degeneration is used as an animal model for AMD, because IS/OS disruption of retinal tissue has been observed in mice treated with NaIO_3_ (Figure 7A) [19,47]. Previous studies have reported endogenous retinal repair following rapid RPE damage induced by relatively high doses of NaIO_3_ (50–100 mg/kg). The administration of 40mg/kg NaIO_3_ has been reported to be sufficient to generate disturbances in retinal function by retinal degeneration in mice and rats [48,49]. In the current study, we observed that the intravenous administration of NaIO_3_ (40 mg/kg) induced RPE degeneration in 7 days (Figure 6). We also found that quercetin treatment ameliorated NaIO_3_-induced retinochoroidal degeneration in vivo.

NaIO_3_-induced RPE degeneration has been shown to lead to the production of pigment-rich substances, and reductions in the thickness of both the outer and inner segments of the photoreceptor and the ONL [18,49,50]. In addition, exposure of RPE cells to NaIO_3_ has been shown to induce a loss of their integrity and cause enlargement [51]. In this study, we found that the thicknesses of the total retina, INL, and ONL of NaIO_3_-treated mice were significantly lower compared with animals in the mock group at day 7, however, quercetin treatment could prevent the retinal degeneration caused by NaIO_3_ (Figure 6 and Figure 7).

Manganese superoxide dismutase (SOD2) is a primary antioxidant located on the matrix side of inner mitochondrial membranes, where it dismutates the superoxide byproducts of oxidative phosphorylation (OXPHOS) to H_2_O_2_, and the latter is further reduced by glutathione peroxidases and catalase [52]. In this study, we demonstrated that NaIO_3_ could induce H_2_O_2_ accumulation due to cell death caused by ROS through enhanced SOD activity and the inhibition of GSH and CAT activity (Figure 3A,B). In addition, we showed that quercetin attenuated the NaIO_3_-induced intracellular cell death caused by ROS via increases in catalase and GSH activity and reduced SOD activity in the ARPE-19 cells. There were a number of limitations to the present study. First, fundoscopy and electroretinogram (ERG) studies were not performed, and these examinations should be conducted in future studies to evaluate retinal function.

**Figure 9:**
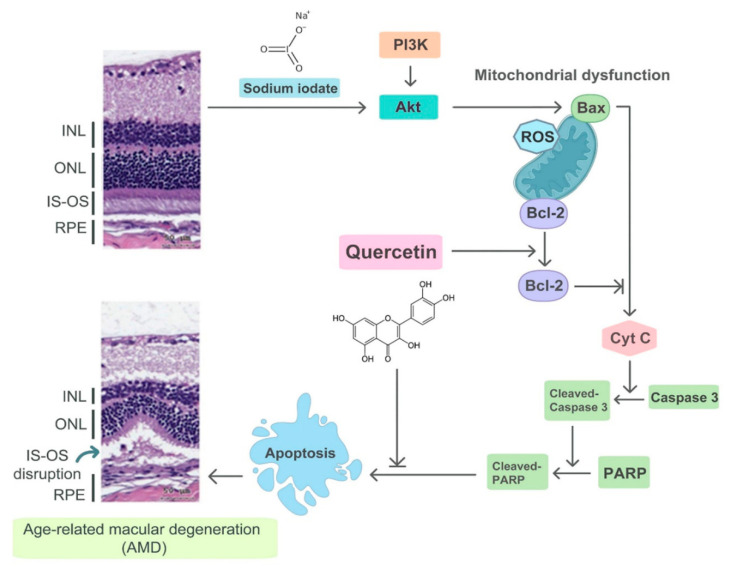
Illustration of the mechanism of NaIO_3_-induced retinal injury and the effects of quercetin. NaIO_3_ induces mitochondrial dysfunction through the PI3K/AKT signaling pathway and then Bax activates Cytochrome C, which causes apoptosis. Finally, IS-OS disruption occurs, which mimics AMD changes within the retinal tissue. Quercetin can activate Bcl-2 and inhibit apoptosis, thereby reducing the accumulation of oxidative damage.

To the best of our knowledge, this is the first study to demonstrate the protective effects of quercetin on NaIO_3_-induced retinal degeneration in a mouse model. To conclude, we found that quercetin reduced NaIO_3_-induced cell apoptosis by downregulating the expressions of Bax, cleaved caspase-3, and cleaved PARP, and upregulating the expression of Bcl-2 via the PI3K/AKT signaling pathway in ARPE-19 cells (Figure 9). Moreover, in vivo experiments demonstrated that quercetin could protect against NaIO_3-_induced retinal damage. We believe that our results will encourage further studies on the potential use of quercetin as a treatment for AMD.

## Data Availability

Not applicable.
